# Comparison of Life Cycle Environmental Impact between Two Processes for Silver Separation from Copper Anode Slime

**DOI:** 10.3390/ijerph19137790

**Published:** 2022-06-24

**Authors:** Zehong Li, Wenbiao Zhang, Bing Xia, Chunying Wang

**Affiliations:** 1 Institute of Geographic Sciences and Natural Resources Research, Chinese Academy of Sciences, 11A Datun Road, Chaoyang District, Beijing 100101, China; lizehong@igsnrr.ac.cn (Z.L.); xiab.16b@igsnrr.ac.cn (B.X.); wangchunying19@mails.ucas.ac.cn (C.W.); 2 College of Resources and Environment, University of Chinese Academy of Sciences, 380 Huaibeizhuang, Huairou District, Beijing 100049, China; 3 Beijing Academy of Social Sciences, 33 North Fourth Ring Middle Road, Chaoyang District, Beijing 100101, China

**Keywords:** copper anode slime, silver separation, life cycle assessment, ecological impact, green manufacturing

## Abstract

The cost of silver separation is lowered when ammonia and hydrazine hydrate are replaced with sodium thiosulfate and sodium dithionite in the process of extracting of metallic silver from copper anode slime. The overall environmental impact of two types of copper silver separation processes from anode slime has been analyzed\using the LCA method. Through the subdivision analysis, we found the raw materials or emission items that should be improved first. The following conclusions are drawn: (1) The life cycle environmental impact of the sodium thiosulfate process is much lower than the existing process; (2) The resource and environmental impacts of the sodium thiosulfate method are mainly in the fields of climate change, photochemical smog, and ionizing radiation, exceeding two-thirds of the impact on all of the resources and environment; (3) In terms of input and output items, the main impact of the new process on the resources and the environment is concentrated on the use of sodium hydroxide, accounting for 33.98% of the total equivalent, followed by sodium thiosulfate and sodium carbonate, respectively. These input–output items are the key fields that need attention in future technology improvement.

## 1. Introduction

Copper anode slime is a by-product of crude copper electrorefining, and a substance that deposits at the bottom of the electrolytic cell during the process of copper electrorefining [1]. The mass is generally about 0.2–1.0% of the anode plate. Anode slime is one of the main raw materials for extraction of rare and precious metals, because it contains a large amount of gold, silver, copper, selenium, tellurium, and platinum group metals [2,3,4,5]. The annual output of copper anode slime in China is about 71,100 tons [6], which is an important source of metallic silver production. With the rapid increase in China’s copper and other non-ferrous metals production [7], the extraction of silver and other precious metals from anode slimes will have increasingly important economic and environmental impacts. The improvement of related extraction technology and its effect on the environment will also attract more and more attention.

The main method to recover silver from anode slime is pyrometallurgy [8,9]. This method usually involves a series of pyrometallurgical processes and consumes a lot of energy [10], and has disadvantages such as high energy consumption, poor silver recovery, and severe air pollution (e.g., SO_2_, lead-containing fumes, arsenic, and antimony) [11]. The wet method and semi-wet method are adopted by most companies in China. The ammonia method is mostly used in the silver separation process—i.e., ammonia and hydrazine hydrate are used as reducing agents to reduce silver ions into elementary substances. However, due to the higher cost and toxicity of hydrazine hydrate, as well as the high volatility of ammonia, the environmental impact is obvious [12].

Semi-hydrometallurgical technology has been developed in order to resolve these disadvantages. The technology includes the following steps: sulfate roasting, sulfuric acid leaching of copper, and sodium sulfite leaching of silver [13,14]. However, this method also has disadvantages, including high consumption of leaching agent and release of harmful gases such as SO_2_. During the pretreatment process, the oxidation of sulfide in the anode slime produces SO_2_ during the sulfation step of baking, thus possibly inducing pollution due to careless operations. During the leaching process, due to its poor stability, the measured Ag (SO_3_)_3_^2−^ is easily oxidized by the oxygen dissolved in the aqueous solution [15]. Thus, it is inevitable to use high-concentration sodium sulfite. As a result, the leaching agent is consumed excessively.

The Institute of Process Engineering of the Chinese Academy of Sciences has proposed an efficient hydrometallurgical technology for recovering silver from anode slime, with sodium thiosulfate as the sliver leaching agent and sodium hydrosulfite for silver reduction. This method has a high recovery rate of silver of up to 95.4% under the optimal conditions. The technology was tested on site by metallurgical enterprise in Jiangxi, with the ammonia method for silver separation, commonly used in China, transformed into a thiosulfate method. With unchanged existing equipment and fine-tuning of the technology process flow, the direct material cost per ton of gold slag dropped from 1654.04 yuan to 802.90 yuan—a decrease of over 50%—thereby achieving good economic benefits. However, the changes in the environmental impact have not yet been analyzed.

There are relatively few studies on the environmental impact of the extraction of precious metals in anode slime. Tang et al. [16] calculated the waste discharge and emission reduction of copper anode slime utilization through joint process analysis. Chai [17] determined the migration process of major pollutants of the waste copper smelting production line via material flow analysis and identified the emission items of the accumulation of pollutants such as smelting slag, smoke, and dust. Nuss [18] analyzed the flow of tellurium in copper anode mud by means of material flow analysis and put forward suggestions on the environmental impact management of tellurium. Iannicelli-Zubiani et al. [19] used the LCA method to analyze the environmental impact of recycling important metals such as copper and gold from electronic waste and identified industrial processes with greater environmental impact. These studies are more static studies on the environmental effects of the production line, lacking comparison of the environmental effects of different processes, and it is therefore difficult to point out the direction of process improvement. From a methodological point of view, direct process analysis and material flow analysis of a production line are limited to the evaluation of the direct environmental impact of the production line. It is difficult to measure the pollution caused by the production of raw materials and waste treatment in the upstream and downstream industries. Relatively, life cycle assessment traces the source of all input and output items, and evaluates its environmental impact more comprehensively.

Life cycle assessment (LCA) is a process of assessing a product, technology, or activity—i.e., an environmental load-related process throughout the entire life cycle, including raw material collection, production, transportation, sales, utilization, recycle, maintenance, and final disposal. Firstly, it identifies and quantifies the consumption and environmental release of energy and materials throughout the life cycle, then it evaluates the impact of consumption and release on the environment, and finally identifies and evaluates the opportunities to reduce the impact [20]. Usually, LCA is divided into four steps: definition of goals and scope determination, inventory analysis, impact evaluation, and improvement evaluation. This method quantifies and evaluates the resource consumption, ecological pressure, and human health impact of specific substances throughout their entire life cycle. Moreover, it further analyzes the impact of differences between the raw materials or products on the environment [21]. Compared with other environmental impact evaluation methods that directly evaluate production, it can more completely assess the entire environmental impact of a specific product or process.

In this paper, life cycle assessment (LCA) method is used to analyze the final resource consumption and pollutant emissions per unit output of the main product—metallic silver—in two different silver separation processes, the ammonia method and sodium thiosulfate method. Then, the impact on different aspects of the ecological environment is analyzed to compare the impact intensity and characteristics between the two processes. On the basis of the overall impact analysis, we analyzed the sensitivity of the environmental impact of all input–output items to select the input–output items that have the greatest ecological impact, and then the direction of technology improvement is proposed. The novelty of this paper is the first life cycle environmental impact assessment of silver extraction from copper anode slime. This assessment will help to identify the links and input–output items with greater environmental impact, and provide direction for future process improvement. The structure of this paper is as follows: Section 2 describes the methodology and data sources used in this paper. Part 3 explains and discusses the analytical results. Part 4 presents final conclusions and suggestions according to the result analyzed, explores the disadvantages, and touches upon a further research direction.

## 2. Methods and Data

### 2.1. Data Sources

The data used in this paper can be divided into foreground data and background data. The former refers to the material and energy data direct input and output in the production process. The latter is the data on the resource consumption and environmental impact of these materials and energy in their respective production processes. The foreground data used in this paper were mainly provided by a company in Jiangxi, China including the data on all input–output items in the two silver separation processes and the data regarding economic value. The background data come from the Gabi database [22,23,24,25,26].

### 2.2. Methods

In this paper, the life cycle assessment method is used to evaluate the final resource consumption and pollutant emissions generated in the two different proceeses of silver separation from copper anode slime. A scope boundary is established for LCA according to the company’s input–output process directly related to silver separation; a life cycle inventory is set up for the production processes according to the data obtained from the field survey; Gabi 9.0 ts is adopted for evaluating the change in the environmental impact of the two silver separation processes using ReCiPe 2016. Different eco-environmental effects—including soil acidification and ozone destruction—are comparatively analyzed at different points of the production processes. Then, the environmental effects of the input and output items of the new process are analyzed, and the input and output substances that have a greater impact on the resources and the environment are found, and the direction for further technological improvement is pointed out. Finally, Monte Carlo analysis is performed on the calculation results to judge the certainty of the calculation results.

#### 2.2.1. Determination of Boundary

The case selected is a metal recycling company located in Jiangxi Province, China. It is a high-tech environmental protection enterprise that specializes in producing black copper, electrolytic copper, nickel sulfate, electrolytic zinc, and precious metals from copper-containing recycling materials such as electronic waste and electroplating sludge; and recovering rare and precious metals, such as gold and silver, from copper anode slime. The company has a designed annual production capacity of 100,000 tons of refined copper and 1500 tons of anode slime, and 90 kg of recovered metallic silver. The production technology is highly representative. This paper introduces the production technology and input–output items of the former ammonia silver separation technology and the new sodium thiosulfate technology developed by the Institute of Process Engineering.

**Figure 1:**
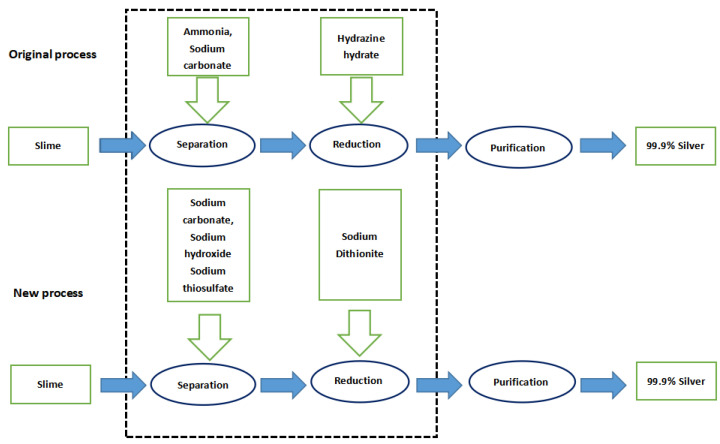
Definition of the LCA scope of the silver separation processes.

The enterprise adopts the ammonia separation technology originally, by which ammonia is used as a leaching agent to generate silver–ammonia complex ions, and hydrazine hydrate as a reducing agent to generate silver. In the new process, sodium thiosulfate is used as leaching agent to generate silver–thiosulfate complex ions, and sodium dithionite is employed as a reducing agent to generate silver. For a simple technology comparison, we took the dominant product—metallic silver—as the benchmark and determined the functional unit of LCA as 1 kg of metallic silver. Figure 1 shows the flow of materials among all the production links in the process of completing a functional unit.

As this paper is focused on the environmental impact generated in the production processes, the ‘cradle-to-gate’ model is selected for the research boundary. That is, from the development of various raw materials to the products, as well as the waste disposal during production, the environmental impact of equipment and infrastructure construction is not taken into consideration as the same facilities are used in both processes. As the silver separation process only produces one beneficial product of metallic silver, the distribution of environmental burdens is not discussed in this paper. The energy consumption rates of the new and old processes are almost the same. Since this paper compares the environmental effects of the new and old processes, the energy consumption can be ignored.

#### 2.2.2. Establishment of Life Cycle Inventory

With Gabi 9.0 ts software (Sphera Solutions GmbH, Leinfelden-Echterdingen, Germany), the direct raw materials used in each production process are associated with the production processes and the emissions to be treated are associated with the treatment process, thereby establishing a complete life cycle inventory. The material input and output in the inventory should be balanced.

The input–output items of original silver separation process include sodium carbonate, liquid ammonia, hydrazine hydrate, and residue of anode slime after gold/copper/selenium separation. The output is metallic silver, residue and wastewater are also generated. Each 1 kg of metallic silver extracted requires 0.95 kg of sodium carbonate, 4.94 kg of ammonia, 4.75 kg of hydrazine hydrate, and 17.09 kg of slime; while 1 kg of crude silver, 15.72 kg of residue, and 11.00 kg of wastewater are produced (Table 1).

In the new process, the input items include sodium hydroxide, sodium carbonate, sodium thiosulfate, sodium dithionite, and metal residue; while the output items remain as metallic silver, residue, and wastewater. Each 1 kg of metallic silver extracted requires 1.52 kg of sodium hydroxide, 0.67 kg of sodium carbonate, 0.91 kg of sodium dithionite, 2.85 kg of sodium thiosulfate, and 17.09 kg of slime, while 13.56 kg of residue and 8.48 kg of wastewater are generated (Table 2).

The quality and consistency of all data input are analyzed. The results show that the data used have high technical and temporal representativeness, and slightly lower location representativeness; the input–output mass difference among different links is less than 0.5%, passing the consistency test (Table 3).

The life cycle inventory analysis could be conducted on the input–output table to acquire data about the classification and sum of the resource consumption and waste emissions generated throughout the production processes. The corresponding data and analysis results are shown in Section 3.1.

#### 2.2.3. Life Cycle Impact Assessment (LCIA)

Through characterization, the impacts of different types of environmental factors are comparatively analyzed and quantified. During the calculation process, characteristic factors are used to convert the results in the life cycle inventory into measurable units for specific environmental impacts. For example, the impact of various substances on global warming is all converted into carbon dioxide equivalent, and various quantitative indicators of ecological environmental impact are obtained for comparison and analysis. Finally, the characterization results of the life cycle are weighted in order to reveal the overall environmental impact. Weighting assigns various weight coefficients based on value selection to different impact type index results and then combines the weighted results.

**Figure 2:**
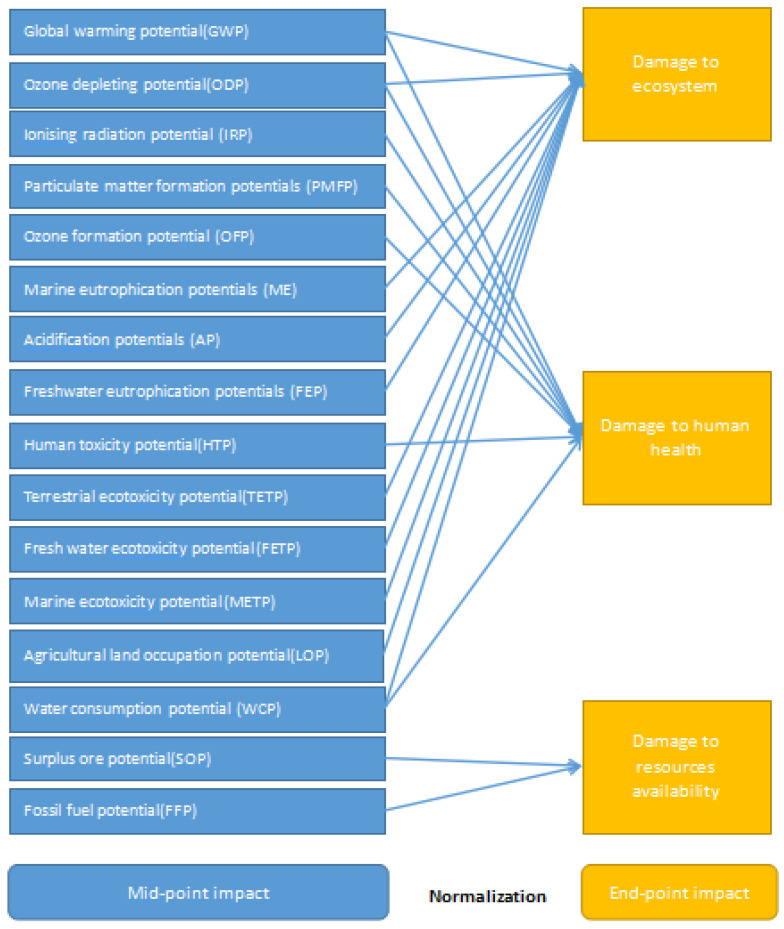
LCA-ReCiPe 2016 life cycle impact assessment process (Huijbregts, Steinmann et al., 2017) [27].

Using LCA-ReCiPe 2016 [27] characterization and normalization method system (mid-point and end-point method) weighted addition, the life cycle list is further converted into impact marks in different areas. ReCiPe 2016 can be regarded as a combine of two models: the mid-point indexes of CML and the end-point indexes of eco-indicator are adopted, so the eco-environmental impact of the production processes can be demonstrated by the evaluation results more comprehensively. Compared with the earlier versions, the parameter setting in ReCiPe 2016 shows significant global applicability. The method considers 16 mid-point indexes. On this basis, the 16 mid-point indexes are converted into three categories of end-point indicators through mid-point-to-end-point conversion. Finally, according to the ratio of each indicator value to the global average value, all indicators are normalized to obtain comparable normalized indicator values (Figure 2).

#### 2.2.4. Uncertainty Analysis

Due to the quality of data collection and the error range, the environmental impact results calculated by the LCA method may be uncertain. This paper will use the Monte Carlo analysis method in Gabi software to evaluate the uncertainty of the calculation results. The mean, standard deviation (SD), and coefficient of variation (CV) are used to quantify the uncertainty. The CV is a parameter that measures the magnitude of the standard deviation relative to the mean. The calculation formula of coefficient of variation is: coefficient of variation CV = (standard deviation/mean) × 100%.

The number of Monte Carlo iterations is set to 1000, and the confidence interval is within 95%.

## 3. Results and Discussion

### 3.1. Results and Discussion of Inventory Analysis

In general, 12,847.50 kg of total materials are required for producing 1 kg of metallic silver by the new process, while 13,402.75 kg of total materials are required for producing 1 kg of metallic silver by the original process. This shows that—calculated by mass—the impact on the resources and environment exerted in the new process is less severe. In terms of itemization, the new process has a lower material flow than the original process except for sea water emission. To be specific, the main energy consumption is all non-renewable energy, while most of the resource consumption is in renewable resources. In terms of emissions, the main emission is freshwater emission, followed by emission to air (Table 4).

### 3.2. Results and Discussion of Impact Assessment

The specific number of 16 eco-environmental indicators of the two processes are shown in the table below. The new process is better than the original process in terms of 13 indexes, including GWP, PMFP, FFP, WCP, FETP, FEP, LOP, ME, SOP, OFP, ODP, AP, and TETP. The global warming effect is 4.61 kg CO_2_ eq., equivalent to 58.06% of the original process; the particulate matter is 0.004 kg PM2.5 eq., equivalent to 49.02% of the original process; the fossil energy consumption is 1.57 kg oil eq., only about 30.43% of the original process. The remaining three indexes—i.e., human toxicity, ionizing radiation, and seawater ecotoxicity—are inferior to the original process, equivalent to 153.45%, 192.31%, and 126.65% of the original process, respectively (Table 5).

**Figure 3:**
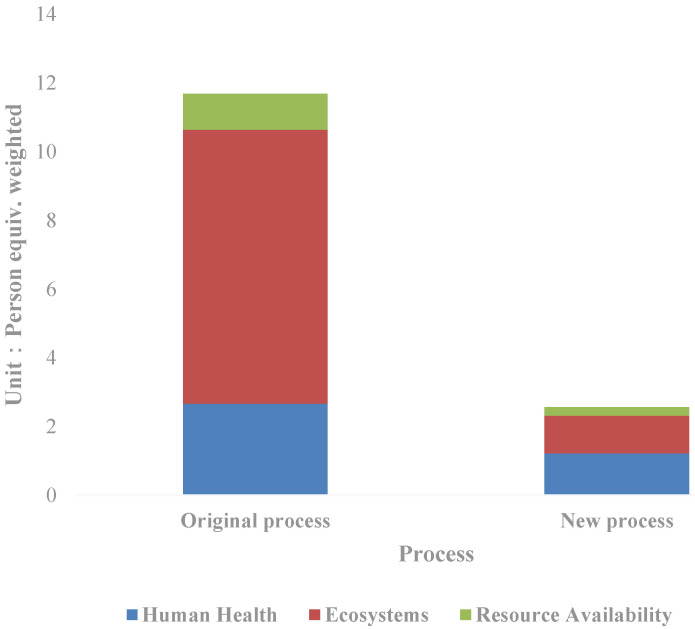
Comparison of the life cycle impact indicators of the silver separation processes.

Through normalization, the total value of the eco-environmental impact is obtained as follows (Figure 3): The weighted person equivalent in the new process is 2.56, equal to 21.94% of that in the original process. To be specific, the human health impact equivalent is 1.22, the ecological impact equivalent is 1.09, and the resource availability impact equivalent is 0.25. The corresponding values of the original process are 2.67, 1.97, and 1.05. Compared with the new process, the ecological impact decreases the most, to only 13.72% of the original process; followed by resource availability, equal to 23.73% of the original process; even as for the human health impact, it decreases by more than half in the new process, equivalent to 45.78% of the original process. From the perspective of the new process itself, the greatest impact on the resources and environment is the human health impact, accounting for 47.06% of the total weighted human equivalent, followed by the ecological impact. Furthermore, the impact is mainly divided into climate change, photochemical smog and ionizing radiation, which account for 37.23%, 17.87%, and 13.38% of the total equivalents respectively. The sum of the three items exceeded two-thirds of the total impact on the resources and environment, which is a key area that needs attention in future technology improvement.

**Figure 4:**
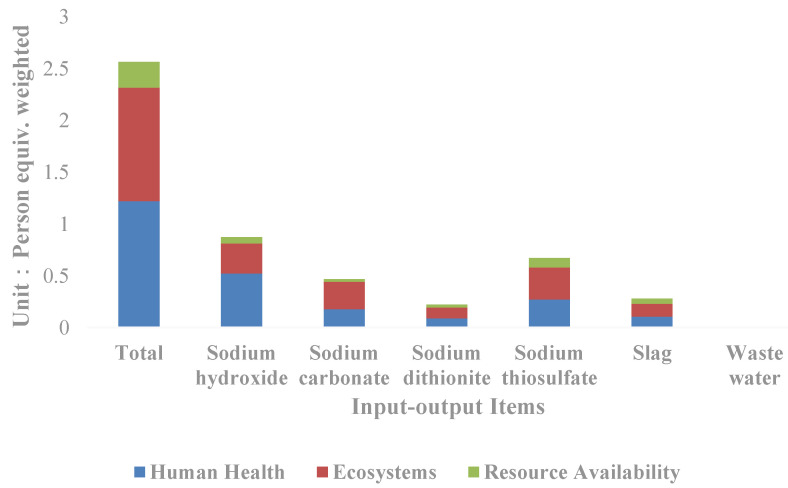
Comparison of the life cycle impact indicators of input–output items of the silver separation processes.

In terms of each input–output item (Figure 4), it can be seen that sodium hydroxide is the input–output item with the greatest impact on the resources and environment, and the weighted person equivalent reaches 0.87, accounting for 33.98% of the total equivalent, followed by sodium thiosulfate and sodium carbonate, which account for 26.10% and 18.30% of the total equivalent respectively. By classification, sodium hydroxide contributes to the greatest impact on human health, accounting for 42.90% of the total human health impact; followed by sodium thiosulfate and sodium carbonate, accounting for 22.25% and 14.66% respectively. Sodium thiosulfate contributes to the greatest impact on the ecological and resource availability, accounting for 28.47% and 34.61% of the total respectively, followed by sodium hydroxide and sodium carbonate.

**Figure 5:**
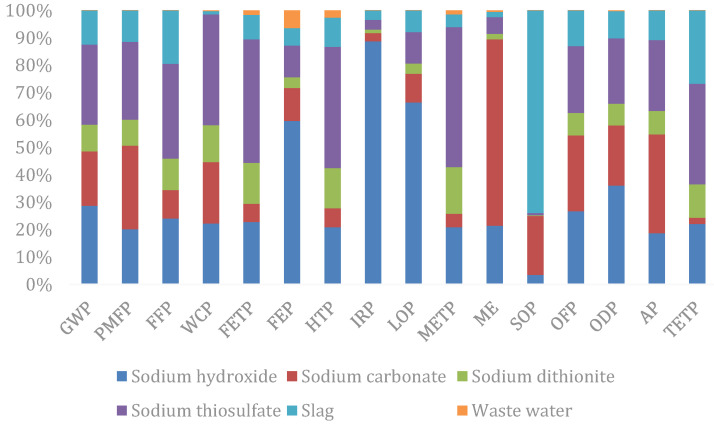
Subdivided end-point indicators of the input–output items of the silver separation processes.

In terms of each impact factor (Figure 5), sodium thiosulfate exerts the greatest impact in the field of GWP, PMF, FFP, WCP, FETP, FEP, METP, OFP, AP, and TETP; sodium hydroxide exerts the greatest impact in the field of ionizing radiation, land use changes, and ozone depletion; solid waste disposal is the largest cause of metal consumption, while wastewater treatment causes the greatest impact on seawater eutrophication.

### 3.3. Uncertainty Analysis

The Monte Carlo analysis module in GaBi software is used to analyze the uncertainty of the calculation results (Table 6). The analysis results show that the CVs of the input–output quality analysis results are 4.61% and 4.60% respectively; that is, the certainty is very significant. In the mid-point data, PMFP (8.79%), FFP (6.83%), METP (6.02%), and TETP (9.37) are significant at the 10% level, and the rest are significant at the 5% level. The CV value of the end-point data is 2.25%, which is less than 5%, indicating that the calculation results have high reliability.

## 4. Conclusions and Recommendation

The life cycle environmental impact of the sodium thiosulfate-based silver separation technology is much lower than that of the original process. The weighted person equivalent is approximately 21.94% of the original process. The ecological impact, human health impact, and resource availability are equal to 13.72%, 45.78%, and 23.73% of the original process respectively. The new process has good environmental and economic benefits and broad application prospects. The new process brings in an additional economic income of 8.12 yuan per kilogram of silver. China has an annual output of 71,100 tons of copper anode slime [6], from which about 4000 tons of metallic silver can be extracted. If the sodium thiosulfate method is adopted for silver separation, an additional economic income of more than 30 million yuan can be generated annually. Meanwhile, greenhouse gas emissions (1.3 × 10^4^ tons of CO_2_ equivalent), fossil energy consumption (1.6 × 10^4^ tons of oil equivalent), acidification (2.5 × 10^3^ tons of sulfur dioxide equivalent), and particulate matter emissions (3.1 × 10^2^ tons of PM2.5 equivalent) can be reduced.

The new process has the greatest impact on human health, accounting for 47.06% of the total weighted human equivalent. Subdivided further, the impact is mainly divided into climate change, photochemical smog, and ionizing radiation, which account for 37.23%, 17.87%, and 13.38% of the total equivalents respectively. The sum of the three items exceeded two-thirds of the impact on total resources and environment. In terms of input and output, the main impact of the new process on the resources and environment is concentrated on the use of sodium hydroxide, accounting for 33.98% of the total equivalent, followed by sodium thiosulfate and sodium carbonate, accounting for 26.10% and 18.30% of the total equivalent respectively. These input and output items are the key areas that need attention in future technology improvement.

In this paper, the overall environmental impacts of two copper anode slime silver separation processes were analyzed using the life-cycle assessment method, and the ultimate resource consumption and pollutant emissions arising from the two silver separation processes were evaluated. Additionally, two types of environmental impact in different areas were analyzed through subdivision analysis, revealing the raw materials or emission items that should be improved first. This conclusion has greater guiding significance for the improvement of the production technology.

This paper has some shortcomings caused by the test progress. First, limited by the shortcoming of technical analysis, this paper fails to further analyze the technology mechanism of the main raw material consumption and waste discharge; second, this article only evaluates the environmental impact of existing production methods, and proposes improvement directions on this basis, but failed to propose a clear technological path for improvement. These issues will be important directions for our further research.

## Figures and Tables

**Table 1:** Material inventory of original silver separation process.

Material	Uint	Quantity	Note
Input			
Sodium carbonate	kg	0.95	
Ammonia	kg	4.94	
Hydrazine hydrate	kg	4.75	
Slime	kg	17.09	Self-produced
Output			
Crude silver	kg	1.00	
Slag	kg	15.72	
Waste water	kg	11.00	

**Table 2:** Material inventory of new silver separation process.

Material	Uint	Quantity	Note
Input			
Sodium hydroxide	kg	1.52	
Sodium carbonate	kg	0.67	
Sodium dithionite	kg	0.91	
Sodium thiosulfate	kg	2.85	
Slime	kg	17.09	Self-produced
Output			
Crude silver	kg	1.00	
Slag	kg	13.56	
Waste water	kg	8.48	

**Table 3:** Data quality assessment of the silver separation processes.

Unit: %	Completely Representative	Partly Representative	Not Representative
Technique	94.4	0	5.56
Location	44.4	50	5.56
Time	94.4	0	5.56

**Table 4:** Life cycle resource depletion and emissions comparison of the silver separation processes.

Unit: kg	Original Process	New Process
**Flows**	13,402.75	12,847.50
**Energy resources**	6.21	2.17
Non-renewable energy resources	6.21	2.17
Renewable energy resources	0.00	0.00
**Material resources**	6700.84	6415.41
Non-renewable resources	34.96	15.18
Renewable resources	6665.83	6400.21
**Emissions**	6695.70	6429.91
Deposited goods	35.63	24.65
Emissions to air	149.85	75.03
Emissions to fresh water	6501.01	6308.29
Emissions to sea water	9.22	21.94
Emissions to agricultural soil	0.00	0.00
Emissions to industrial soil	0.00	0.00

**Table 5:** Comparison of the life cycle impact indicators of the silver separation processes.

Factor	Unit	Original Process	New Process
GWP	kg CO_2_ eq.	7.94	4.61
PMFP	kg PM2.5 eq.	8.16 × 10^−2^	4.00 × 10^−3^
FFP	kg oil eq.	5.75	1.75
WCP	m^3^	2.56 × 10^−2^	1.90 × 10^−2^
FETP	kg 1,4 DB eq.	7.98 × 10^−4^	7.29 × 10^−4^
FEP	kg P eq.	2.44 × 10^−5^	1.28 × 10^−5^
HTP	kg 1,4-DB eq.	1.16 × 10^+1^	1.78 × 10^+1^
IRP	kBq Co-60 eq. to air	3.12 × 10^−1^	6.00 × 10^−1^
LOP	Annual crop eq.·y	2.11 × 10^−1^	9.08 × 10^−2^
METP	kg 1,4-DB eq.	6.98	8.84
ME	kg N eq.	3.59 × 10^−4^	1.80 × 10^−4^
SOP	kg Cu eq.	9.71 × 10^−2^	7.29 × 10^−2^
OFP	kg NOx eq.	2.72 × 10^−2^	2.12 × 10^−2^
ODP	kg CFC-11 eq.	3.43 × 10^−5^	1.71 × 10^−6^
AP	kg SO_2_ eq.	6.47 × 10^−1^	1.26 × 10^−2^
TETP	kg 1,4-DB eq.	2.23	1.76

**Table 6:** Monte Carlo analysis of the results of the silver dividing processes LCA.

	Unit	Mean	CV
Mass-input	kg	1.25 × 10^+4^	4.61%
Mass-output	kg	8.60 × 10^+1^	4.60%
Mid-point-GWP	kg CO_2_ eq.	7.50 × 10^+3^	4.84%
Mid-point-PMFP	kg PM2.5 eq.	1.28 × 10^+4^	8.79%
Mid-point-FFP	kg oil eq.	1.53	6.83%
Mid-point-WCP	m^3^	3.72 × 10^−2^	4.61%
Mid-point-FETP	kg 1,4 DB eq.	1.92 × 10^+2^	3.58%
Mid-point-FEP	kg P eq.	2.94 × 10^+4^	4.46%
Mid-point-HTP	kg 1,4-DB eq.	9.12 × 10^+2^	3.17%
Mid-point-IRP	kBq Co-60 eq. to air	3.01 × 10^+2^	2.40%
Mid-point-LOP	Annual crop eq.·y	1.58 × 10^+4^	3.04%
Mid-point-METP	kg 1,4-DB eq.	5.38 × 10^−1^	6.02%
Mid-point-ME	kg N eq.	1.70 × 10^+2^	2.98%
Mid-point-SOP	kg Cu eq.	2.42 × 10^+1^	4.48%
Mid-point-OFP	kg NOx eq.	2.39 × 10^+1^	3.94%
Mid-point-ODP	kg CFC-11 eq.	3.60 × 10^−2^	3.65%
Mid-point-AP	kg SO_2_ eq.	6.62 × 10^+2^	3.65%
Mid-point-TETP	kg 1,4-DB eq.	3.99 × 10^+3^	9.37%
End-point	Weighted person equivalents	1.25 × 10^+4^	2.25%

## Data Availability

The data presented in this study are available on request from the corresponding author. The data are not publicly available due to business secrets of enterprises.
